# Leveraging User-Friendly Network Approaches to Extract Knowledge From High-Throughput *Omics* Datasets

**DOI:** 10.3389/fgene.2019.01120

**Published:** 2019-11-13

**Authors:** Pablo Ivan Pereira Ramos, Luis Willian Pacheco Arge, Nicholas Costa Barroso Lima, Kiyoshi F. Fukutani, Artur Trancoso L. de Queiroz

**Affiliations:** ^1^Center for Data and Knowledge Integration for Health (CIDACS), Instituto Gonçalo Moniz, Fundação Oswaldo Cruz, Salvador, Brazil; ^2^Laboratório de Genética Molecular e Biotecnologia Vegetal, Centro de Ciências da Saúde, Universidade Federal do Rio de Janeiro, Rio de Janeiro, Brazil; ^3^Departamento de Bioquímica e Biologia Molecular, Universidade Federal do Ceará, Fortaleza, Brazil; ^4^Multinational Organization Network Sponsoring Translational and Epidemiological Research (MONSTER) Initiative, Fundação José Silveira, Salvador, Brazil

**Keywords:** correlation networks, graph, high-throughput sequencing, network analysis, omics, protein–protein interaction, regulatory networks, systems biology

## Abstract

Recent technological advances for the acquisition of multi-*omics* data have allowed an unprecedented understanding of the complex intricacies of biological systems. In parallel, a myriad of computational analysis techniques and bioinformatics tools have been developed, with many efforts directed towards the creation and interpretation of networks from this data. In this review, we begin by examining key network concepts and terminology. Then, computational tools that allow for their construction and analysis from high-throughput *omics* datasets are presented. We focus on the study of functional relationships such as co-expression, protein–protein interactions, and regulatory interactions that are particularly amenable to modeling using the framework of networks. We envisage that many potential users of these analytical strategies may not be completely literate in programming languages and code adaptation, and for this reason, emphasis is given to tools’ user-friendliness, including plugins for the widely adopted Cytoscape software, an open-source, cross-platform tool for network analysis, visualization, and data integration.

## Introduction

The analysis of high-throughput datasets using the framework of networks has gained widespread adoption in the biological sciences. With approaches in this field shifting from a mostly reductionist perspective towards a more holistic view of natural phenomena (Barabási and Oltvai, 2004; Berlin et al., 2017), the analytical tools used to extract knowledge from data have also adapted. The vocabulary of networks is particularly suitable for studying problems that explicitly focus on the *relationships* among elements, where the latter can be any entity under study, including but not limited to genes, transcripts, proteins, or metabolites. With sheer amounts of data that can be obtained from instruments such as high-throughput sequencers, analytical strategies that permit broader insights of the functional roles of each element are warranted, and this can be achieved by the use of network approaches.

In this Review, we focus on the various uses of network methods to the analysis of large-scale *omics* datasets, which are those generated using medium- and high-throughput technologies in genomics, transcriptomics, proteomics, and metabolomics experiments. First, key concepts and terminology of this area are presented, followed by the introduction of biological network variants, namely correlation networks (*Correlation networks allow disclosing of relevant associations in omics datasets*), gene regulatory networks (GRNs) (*Gene regulatory networks permit an improved understanding of the cell’s transcriptional circuitry*), and protein–protein interaction (PPI) networks (*Protein–protein interaction networks provide an integrated view of the proteome’s organization and interactions*). Methods to perform key analysis in a network are presented in *A primer on network analysis and visualization*. With every approach, computational tools that we considered both appropriate and user-friendly are presented. User-friendly tools were defined as those that provide a point-and-click graphical user interface, which does not mean that they have limited functionality or that they are only used by those without extensive programming literacy. Rather, they can be used to complement analyses performed in different environments, such as R or Python scripts, and usually offer improved layouts and visualization modes compared to less friendly alternatives. Our Review differs from that of others who have engaged in similar challenges (for instance, the works of Aittokallio and Schwikowski, 2006; Stevens et al., 2014; Huang et al., 2017), since we primarily target the non-programmer who wants to apply network methods to a dataset of interest. Luckily, network analysis is an area that has greatly benefited from the existence of excellent analysis software such as Cytoscape (Shannon et al., 2003) (https://cytoscape.org/), Gephi (Bastian et al., 2009) (https://gephi.org), and NAViGaTOR (Brown et al., 2009), to name a few. Gephi and Cytoscape, in particular, can be extended by the many plugins created by third-party developers and available in official repositories (Saito et al., 2012), and these were at the heart of the current review. While the aforementioned types of networks are widely employed, there are many other applications that are not in the scope of this work. As an example, the modeling of (bio)chemical networks using graph–theoretic approaches have advanced our understanding of bacterial and eukaryotic metabolism (Klein et al., 2012; Dutta et al., 2014; Jha et al., 2015), and were the object of previous reviews (see, e.g., Lacroix et al., 2008; Cottret and Jourdan, 2010). Biology and Biomedicine are, indeed, areas which have been greatly benefited by the use of network techniques resulting from cross-pollination among disciplines.

### Beyond the Empirical, Towards Formalism: What Are Networks?

Network is a general term used in many different contexts: social networks, traffic networks, ecological networks, computer networks, among others, all share a common theme related to the interaction among a set of disparate elements, viz. people, vehicles, species, and computers. The topology of networks and the interactions within can be formally studied from a graph–theoretic viewpoint, which allows for a mathematical representation and formalism, while also facilitating visualization of the network. Since several distinct graph representations exist, for generality we will focus on the description of simpler types of graphs. In general, a graph Γ = (*V,E*) is composed of a finite set *V* of nodes (or vertices), and *E* of (directed or undirected) edges (or links). In the case of *omics* datasets, each node *v* ∈ *V* could represent a (bio)chemical entity such as a gene, transcript, protein, or metabolite, and an edge *e* = {*v*_1_,*v*_2_} ∈ *E* exists between two nodes when there is evidence for their interaction, which in turn depends on the specific aim of the modeled network, which guides the definition of interaction. For instance, in the simplest type of correlation network, one could specify a hard threshold over all pairwise values of Pearson’s correlation coefficients in order to determine whether any two nodes are connected. On the other hand, in a PPI network, edges between protein nodes exist when evidence for their physical interaction is available, which could be obtained by a wealth of techniques that include co-immunoprecipitation, affinity purification, proteomics, and computational approaches (Ngounou Wetie et al., 2014).

**Figure 1:**
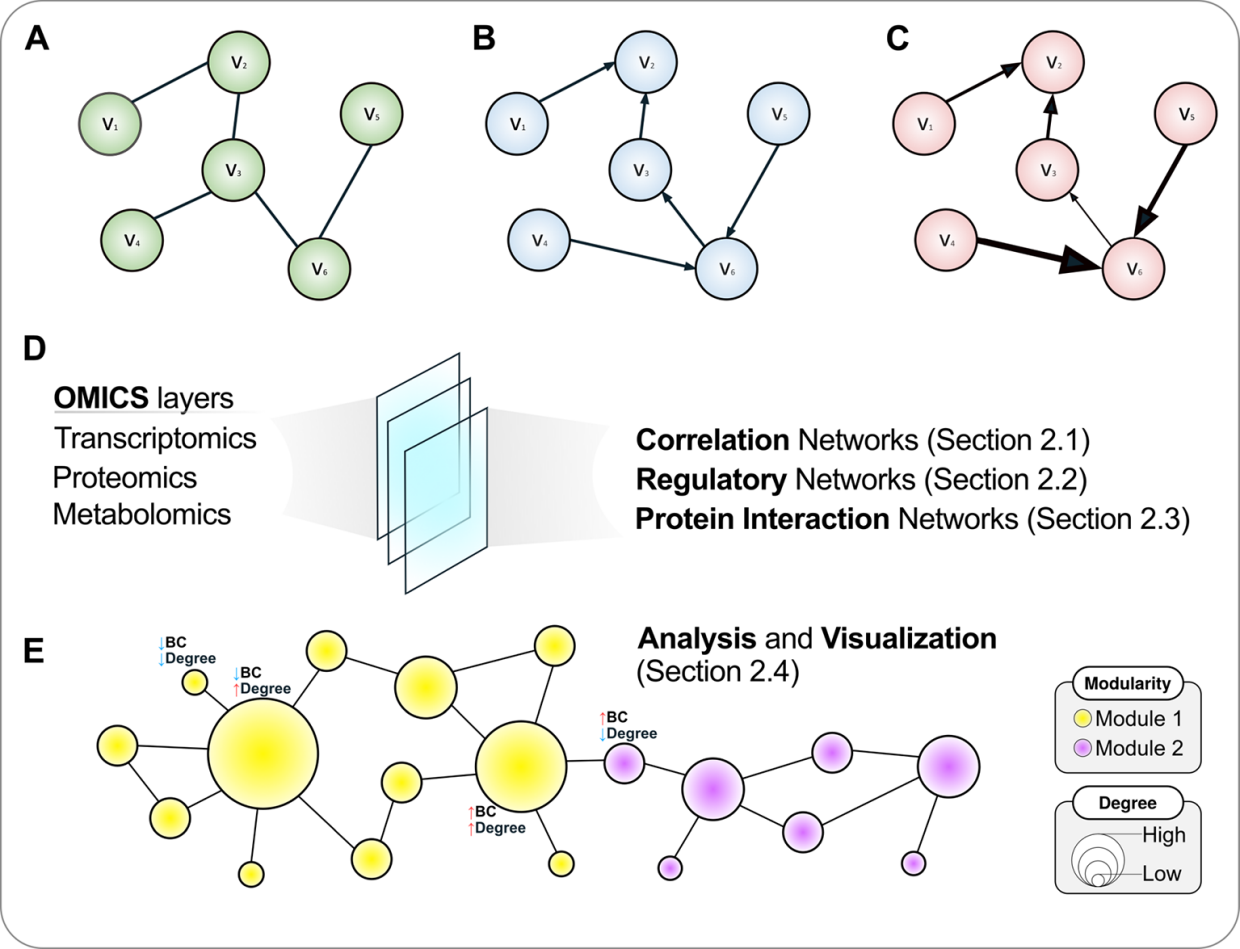
A roadmap to network concepts covered in this review. Three simple six-node graphs are shown in the upper panel. These graphs can be undirected **(A)**, directed **(B)** or weighted directed **(C)**. In the latter, the thickness of edges reflects the weights of the interactions. Various *omics* datasets can be analyzed using the language of networks, which are discussed in the following sections **(D)**. **(E)** Once a network is attained, further analyses are warranted, which include disclosing modules or communities and calculating topological metrics such as node degree and betweenness centrality (BC), covered in *A primer on network analysis and visualization*. The size of a node is proportional to its degree, while the color reflects the community structure in this illustrative example where two modules are disclosed. For selected nodes, interpretations of node BC and degree are presented.

The edges in a graph can be undirected (**Figure 1A**) or directed (**Figures 1B, C**). In directed graphs, there is a specific sense pointing at the direction of a given interaction, such as a transcription factor (TF) that regulates a given gene in a regulatory network (a causal relationship), while undirected graphs describe two-way associations such as the co-expression of genes in a correlation network, in which a significant correlation *per se* does not provide sufficient evidence to infer whether any of the compared genes regulates or is being regulated by the other, or even by an upstream regulator acting on both simultaneously. That is, correlation does not imply causation, and hence the undirected graph is a more appropriate representation of this relationship.

Graphs can also have numerical weights associated with each interaction, the interpretation of which depends on the specific application under study (**Figure 1C**). In a correlation network, for instance, weights could represent the magnitude of the correlation statistic. Also possible is to set weights based on the confidence of the interaction as measured by a relevant parameter. As an example, the STRING database (http://string-db.org), which harbors information on physical and functional PPIs, quantifies interaction weights between proteins as a combined score dependent on the nature (experimental or computational prediction) and quality of the supporting evidence (Szklarczyk et al., 2017). **Table 1** summarizes the biological interpretation of nodes, edges, and edge weights for the three types of networks considered in this study. While these interpretations are typical for these kinds of biological networks, studies may employ different analytical strategies that lead to variations on how to account edge directionality or weights, for instance. As an example, regulatory networks are usually inferred using a bipartite graph representation, where nodes are of two different types (either a TF or a target gene). In this case, edge directionality characterizes an underlying regulatory event (activation or inhibition) of a TF towards a target gene, hence these networks are usually modeled as a directed graph (Narasimhan et al., 2009; Song et al., 2017).

**Table 1:** Biological interpretation of nodes, edges, and edge weights for the *omics*-derived networks under study.

Type of network	Graph representation	Edge directionality	Biological interpretation of		
			nodes	edges	edge weights
Correlation network	Simple graph	Undirected	Genes, proteins, or metabolites	Correlation (co-expression) between a pair of biological entities, which is calculated from a measure of abundance, such as gene expression or metabolite concentration	The strength of correlation (co-expression) between the pair of nodes
Gene regulatory network	Simple or bipartite graphs	Usually directed	Genes in the simple graph; transcription factors and target-genes in the bipartite graph	A regulatory relationship	The degree of the regulatory relationship
Protein–protein interaction network	Simple graph	Usually undirected	Proteins	The direct contact (physical binding) between proteins, but can represent indirect (functional) interactions between the peptides	Usually unweighted, but can be valued to represent the support (confidence) for a given interaction

## How to Disclose Networks From High-Throughput *Omics* Datasets

In the following sections, we review and discuss methods to construct various types of networks using a wealth of *omics* datasets as input (**Figure 1D**). While many different computational methodologies to achieve the construction of a network exist, we focus on those that we considered more apt for users without a computational background, especially those that are based on plugins for the popular software Cytoscape (Shannon et al., 2003), which allows visualization, rendering, and analysis of networks in the same computational environment, with the advantage of being open-source, platform-independent, and continuously updated. Once the tools to build these biological networks are covered, we shift our focus towards analysis and visualization aspects of graphs, which are presented in *A Primer on Network Analysis and Visualization* (**Figure 1E**).

### Correlation Networks Allow Disclosing of Relevant Associations in *Omics* Datasets

Recent advances in high-throughput technologies have increased our capacity to assess the elements in different *omics* layers, allowing their simultaneous treatment in single grouped mechanisms that together explain biological events (Carpenter and Sabatini, 2004; Vella et al., 2017). In this sense, the processes that allow for life maintenance in cells can be regarded as an intricate web of complex relationships between molecules such as proteins, lipids, metabolites, and nucleic acids (RNA and DNA) (Barabási et al., 2011). Correlations are arguably the dominant way to infer relationships not only between the elements in these distinct layers of information but also within each layer, as it allows simultaneously examining the associations that drive an observed biological effect, and there are several ways of calculating correlation coefficients. Statistically, the correlation is a measure of the two-way linear association between a pair of variables (Mukaka, 2012). The correlation coefficient permits estimating the degree or strength of this association. The most common and classic correlation statistic is the Pearson’s correlation coefficient (or *r*), which measures linear associations between two variables under the assumption that the data be normally distributed and that observations are independent (Walter and Altman, 1992). Non-parametric methods based on ranks avoid the assumption of normality and are preferred when the data is ordinal, skewed, or presents extreme values (outliers). One such method is the Spearman correlation coefficient, which is a calculation of Pearson’s correlation coefficient on the ranks of the observations, rather than on the raw data, and yields an *r* *_S_* statistic (also called ρ, rho). The Kendall rank correlation coefficient (also called τ, tau) uses the number of concordant and discordant rank pairs to evaluate association. The biweight midcorrelation is less prone to outlier influence because it is a median-based estimation and, like the two previous, yields a robust measurement of association, with the drawback that few tools are available that calculate this metric (Langfelder and Horvath, 2012). Correlation coefficients (*r*, *r* *_s_*, ρ, or τ) are a dimensionless quantity ranging from -1 to 1, where values close to zero indicate no (linear) association whilst values equal to or near 1 (or -1) indicate strong, positive (or negative) correlations, although absolute values as low as 0.3 can already be considered a weak correlation depending on the context (Mukaka, 2012).

Since the relationships between genes, proteins, metabolites and biological entities in general are complex and often nonlinear, while having distributions that can be non-normal, alternative measurements of association are often required (Hardin et al., 2007), and include information-theoretical measures such as mutual information (MI). MI quantifies the dependence between a pair of random variables and, based on the concept of entropy, estimates how much knowledge is gained about a variable (say, expression values of a gene *X*) by observing a second variable (say, expression values of a gene *Y*), hence its name. The MI is zero when the variables are statistically independent, while a positive value denotes a degree of dependence (Steuer et al., 2002). In a scenario of statistical independence, the distribution of values of variable *X* is not altered at all when those of variable *Y* changes. It is worth noting that traditional association measures that disclose only linear relationships are insufficient to reveal statistical independence, exactly because there can be non-linear relationships in the data that these methods do not adequately capture. We refer the reader to the review of de Siqueira Santos et al. (2014) on statistical dependency identification, who further provide illustrative biological examples and simulations using various association statistics.

**Figure 2:**
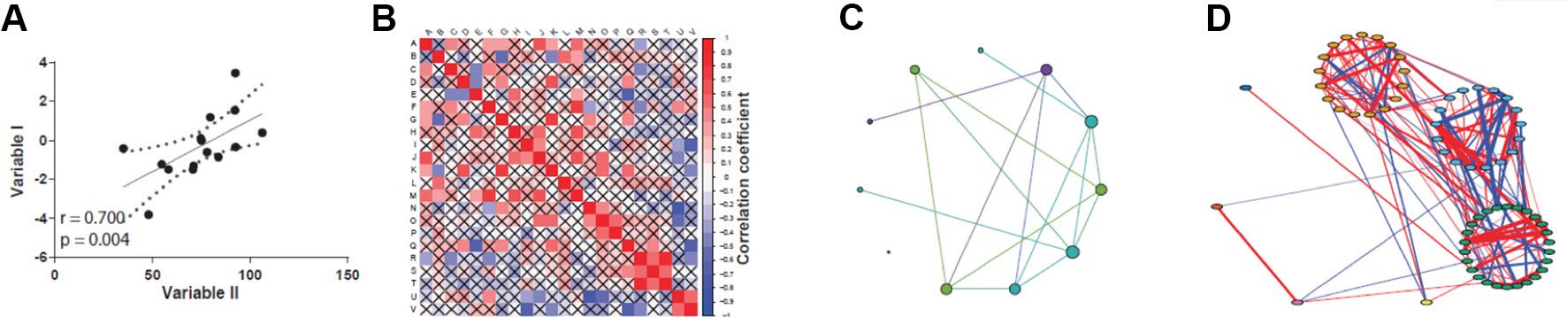
Different views on assessing correlations. **(A)** Classic scatter plot with correlation curve (straight black line). **(B)** Correlation matrix plot, designed with the *corrplot* package (Wei and Simko, 2017). **(C)** Circular layout correlation network, designed with Gephi (Bastian et al., 2009). **(D)** Complex correlation network with modularity coloring, designed with *qgraph* package (Espkamp et al., 2012).

Correlations can be visually assessed by plotting the data as a scatter plot fitted by a line, where the further the data lie from the straight line, the weaker the correlation (**Figure 2A**). While this approach is feasible when few variables are compared, it has limited practicality when dealing with large-scale *omics* datasets, such as high-throughput expression profiling and proteomics. In these cases, methods that create correlation networks are preferred (Zhang and Horvath, 2005; Langfelder and Horvath, 2008; Vella et al., 2017). Once a correlation (or other association statistic) matrix is attained (**Figure 2B**), a network can be inferred (**Figure 2C**). A co-expression network is a particular case of correlation network constructed using genome-wide expression data, although the term is sometimes used to refer to networks created by correlating the abundance of protein or metabolites in proteomics and metabolomics studies. In this network, the nodes are elements such as genes, proteins, or metabolites, and an undirected edge connects a pair of nodes if the correlation statistic between them exceeds a given threshold (**Figure 2C**). This “hard-threshold” approach represents the simplest form of inducing a network from *omics* data, and is limited by the arbitrary nature of the threshold used, which will dismiss slightly undervalued correlations that could be potentially relevant. An alternative, more sophisticated approach to disclose co-expression networks is by using soft-thresholding approaches, of which the weighted gene co-expression network analysis (WGCNA) algorithm is among the most widely employed methods (Langfelder and Horvath, 2008). The main advantage of the WGCNA approach is that no arbitrary thresholding on the correlation values is enforced, which effectively preserves the continuous nature of the correlation distribution. In addition, it is not impacted by the arbitrariness of hard-thresholding methods. In WGCNA, once all pairwise correlations are calculated, an adjacency matrix, which holds information on edge strengths, is obtained by applying a power transformation of the form *f*(*x*) = *x* *^β^*, where *x* are correlation values and β is the soft-thresholding parameter, a positive value set by the user such that the resulting network presents an approximately scale-free property while maintaining high connectivity (see **Box 1** for a primer of important network definitions). As a result, high correlations are emphasized at the expense of low correlations, but without the need of setting an explicit threshold on the correlation values themselves.

Box 1 **Key concepts applied to biological networks**

Biological networks are composed of nodes that can represent different bioentities and have different biological importance for a given network. Regardless of the network size, shared commonalities exist between different biological networks, which allow their comparison. The concepts below describe some characteristics of biological networks and different metrics for topological evaluation of nodes, allowing for prioritization of important elements in the network.

**Scale-free**. A network is considered scale-free when its degree distribution follows a power law. Thus, it is characterized by the presence of many small-degree nodes together with a few highly connected nodes (or hubs), forming an inhomogeneous network. Many biological networks exhibit the scale-free property, including protein interaction and gene co-expression networks.

**Small-world**. When networks exhibit a low number of node intermediates separating any two nodes in the network (*ie.*, low average distance), it is considered a small-world network.

**Modularity**. Biological networks tend to form modules, or clusters of highly connected nodes (**Figure box A**). Modularity takes values between -1 and 1 and reflects the link density within a module as compared to links between modules. In biological networks, nodes with similar functions have a bias to form functional modules.

**Figure Box:**
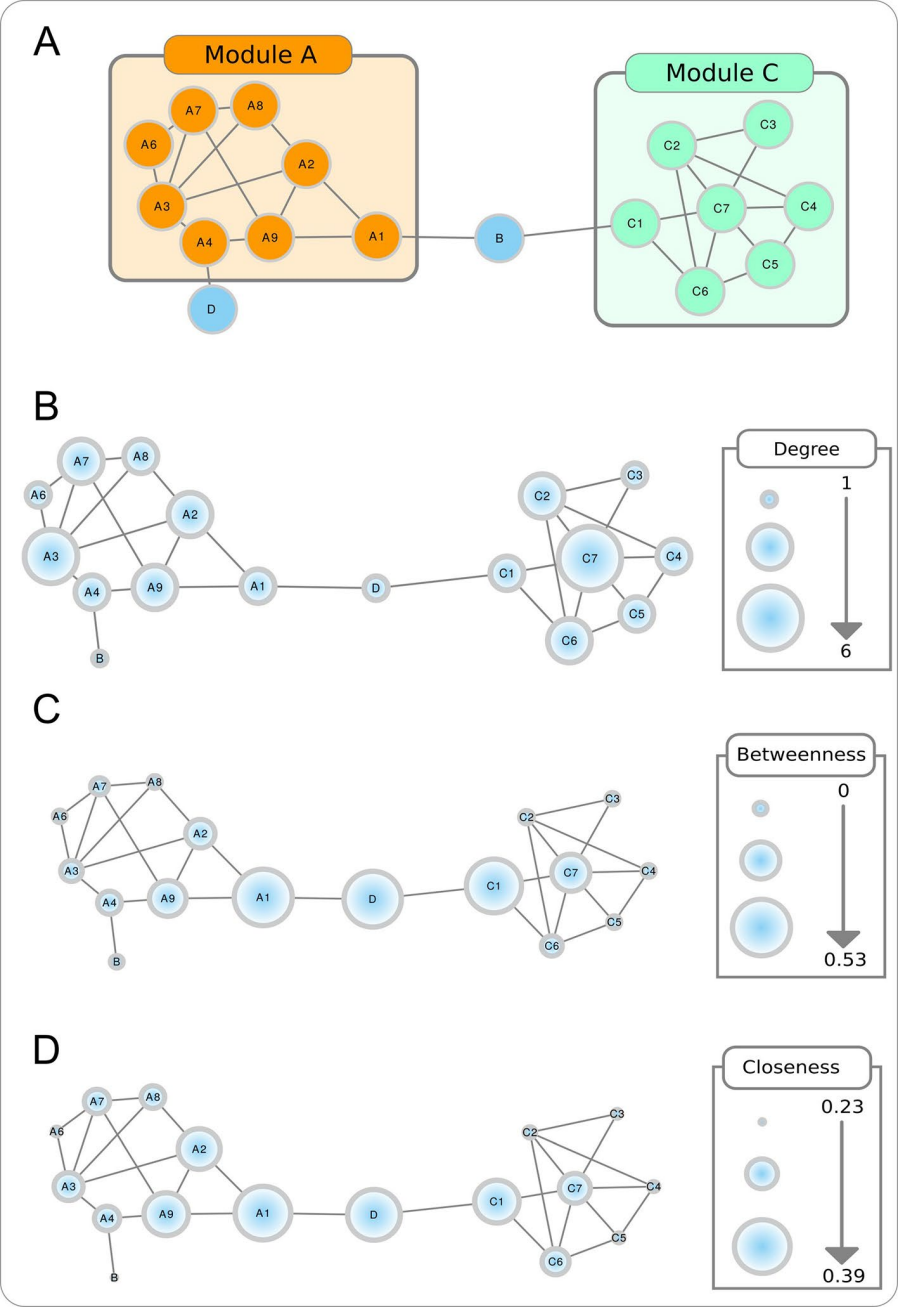
Topological properties of a toy network. The modular aspect of the network is apparent in **A**, with two modules (or partitions) shown. The size of the nodes in **B**–**D** are proportional to, respectively, the node degree, betweenness centrality, and closeness centrality.


**Hubs**. The most highly linked nodes in a network are called hub nodes, which play an important role in defining network scale-freeness. The term is also used to refer to nodes that display high centrality as measured using a relevant metric (see below).

**Shortest (or geodesic) path**. A shortest path is the minimum series of edges that should be traversed to connect two nodes in a network. In a weighted graph, it is the path lending to the minimum sum of edge weights between a node pair.

**Node centrality metrics**

Each component of a network presents topological characteristics that can be translated into biological knowledge and help establish the identification of relevant nodes:

**Node degree**. Refers to the number of nodes directly connected to a specific node, and is obtained by counting the number of interactions that a specific node has with other nodes in the network (**Figure box B**). When the network is directed, this is separated into out-degree (the number of outgoing links from a node) and in-degree (the number of ingoing links in a node). The higher is the degree of a node, the higher will be the probability that it is a hub. Nodes with high degree centrality have more influence on the structure and functionality of a network than nodes with a low degree.

**Betweenness centrality**. Measures the importance of a node to the connection of different parts of a network (**Figure box C**). The betweenness centrality for a node is the proportion, among all shortest paths, of those that use the given node as intermediate. Nodes with these characteristics are usually referred as bottlenecks and can also be considered hubs.

**Closeness centrality**. Measures how close a node is to all the other nodes in the network (**Figure box D**). It is calculated by the reciprocal sum of all shortest paths to all other nodes of the network. The higher the closeness centrality for a node, the closer is the relationship with the remaining nodes in the network.

#### User-Friendly Tools for Constructing Correlation Networks

**Figure 3:**
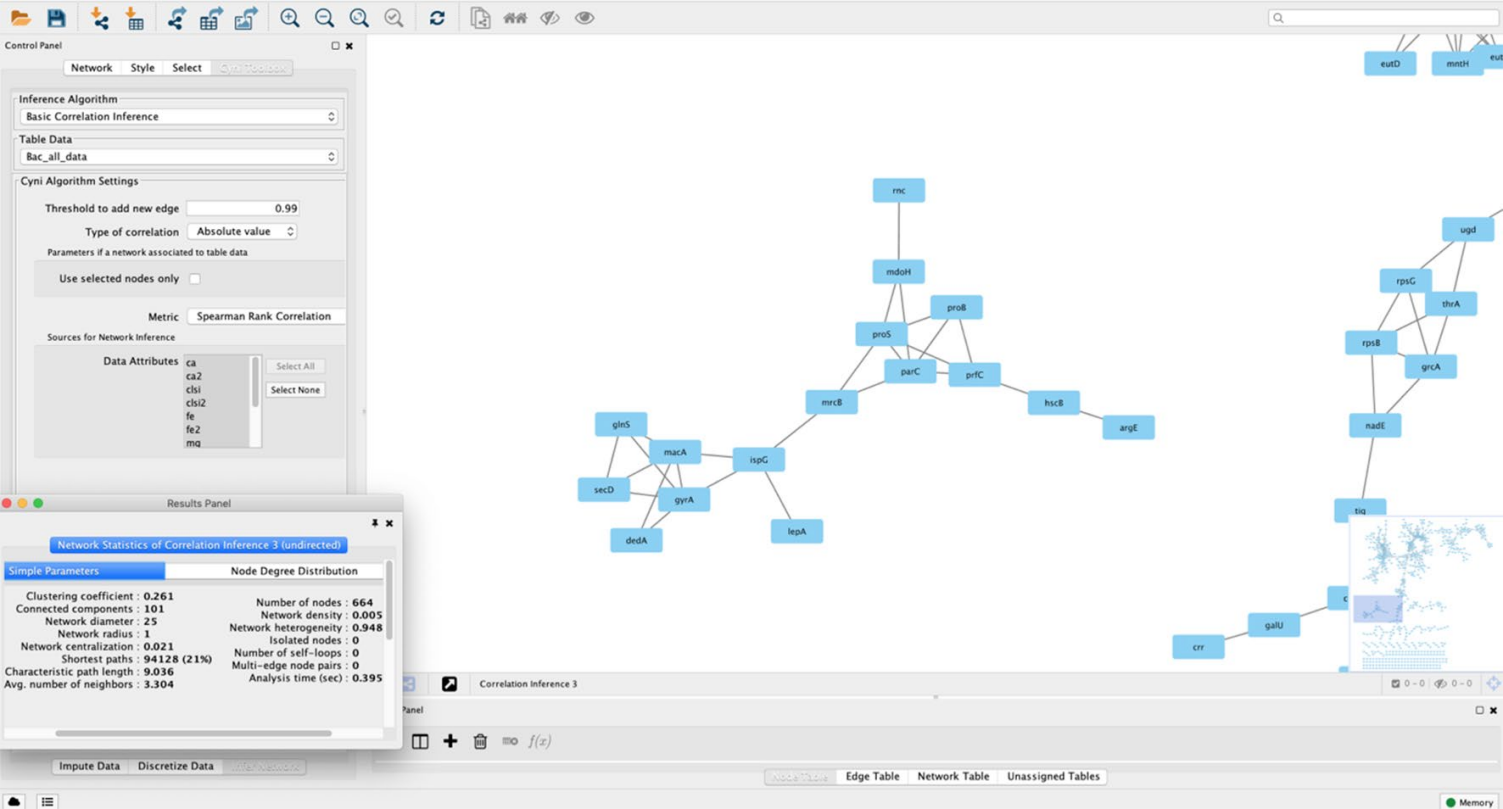
A correlation network constructed using Cytoscape 3.2. The network was built using a bacterial expression dataset, and nodes represent annotated genes, with edges connecting nodes if they pass a correlation threshold calculated using Spearman’s rank correlation in the Cyni Toolbox. In the picture a pop-up menu with the calculated network metrics (using the NetworkAnalyzer plugin in Cytoscape) is shown. Besides the network zoom, the program also shows the whole network in the lower-right screen, as a miniature.

Gene/protein correlation network analysis can be performed using in-house scripts and packages for general-purpose programming languages such as R, Python, Perl, or Java. However, alternatives exist for the bioinformatics user that wants to apply such methods to their data in the absence of a solid computational background (**Table 2**). One of them is based on the Cytoscape environment, which also allows for installing third-party plugins. A specific app developed for correlation network analysis, the *ExpressionCorrelation* app (available at http://apps.cytoscape.org/apps/expressioncorrelation), presents a Pearson’s correlation-based solution. Thus, a table of gene/protein/metabolites measurements is the input and Cytoscape can generate the gene and sample correlation network. This plugin has been applied to the construction of many networks, exemplified by an *Anopheles* gene co-expression network (Shrinet et al., 2014), a correlation network from *Aspergillus* metabolites highlighting those significantly associated to anticancer and antitrypanosomal bioactivity (Tawfike et al., 2019), and co-expression networks from cancer datasets (Wang et al., 2016b; Zhang et al., 2016). Pearson’s correlation statistic, however, presents several limitations as pointed out in the previous section. The Cyni toolbox app circumvents this difficulty by allowing calculation of rank-based correlations such as Spearman’s and Kendall’s, in addition to Pearson’s coefficient (Guitart-Pla et al., 2015). **Figure 3** shows a bacterial co-expression network constructed using Cyni.

**Table 2:** User-friendly computational tools for inferring correlation networks.

Tool	Description	Platform	Reference/URL
Cyni toolbox (Cytoscape)	Performs several correlation analyses and includes other networks inference algorithms.	Multi	http://apps.cytoscape.org/apps/cynitoolbox; (Guitart-Pla et al., 2015)
Expression Correlation app (Cytoscape)	Performs Pearson correlation analysis and network inference.	Multi	http://apps.cytoscape.org/apps/expressioncorrelation
ARACNe/Mutual Information (geWorkbench)	Creates a network based on Mutual Information.	Multi	http://wiki.c2b2.columbia.edu/workbench/index.php/Home; (Floratos et al., 2010)
webCEMiTool	Performs comprehensive modular analyses in a fully automated manner, generating co-expression networks based on the WGCNA method.	Webserver	https://cemitool.sysbio.tools/; (Cardozo et al., 2019)

Another user-friendly solution is *geWorkbench* (Floratos et al., 2010). This tool is an open source Java desktop application that allows correlation using an ARACNe (mutual information-based) implementation (Margolin et al., 2006a), and is particularly suitable for finding regulatory networks from transcriptomic data. In addition, the workbench allows for parameter estimation and is fairly flexible for user customization. Its advantages over the Cytoscape *ExpressionCorrelation* app include the possibility of p-value threshold modification and correction, as well as bootstrap resampling. Thus, the program permits evaluating the statistical significance of the network and keep the more robust associations. However, the user-friendly advantage is not without its costs: the plugin is limited to the calculation of regular correlations (Pearson’s and Spearman’s) and mutual information. Also, the use of more robust correlation statistics, such as the biweight midcorrelation, still requires proficiency in programming languages/R packages, since so far there are no alternatives that incorporate this measure.

The construction of weighted networks using the soft-thresholding approach employed by WGCNA requires the execution of a multi-step pipeline implemented as an R package (Langfelder and Horvath, 2008), thus requiring programming skills to correctly adapt and parametrize the functions and the dataset itself. To circumvent this need, a webserver adaptation of the WGCNA method was recently published as *webCEMiTool*, allowing an user-friendly approach to disclose a weighted co-expression network, detect modules therein, and produce publication-quality visualizations (https://cemitool.sysbio.tools/) (Cardozo et al., 2019). In this context, modules are considered as groups of genes with similar expression profiles, which tend to have related biological functions or be under the influence of the same transcriptional regulator, but a more ample discussion of modularity is presented in *A primer on network analysis and visualization*. *webCEMiTool* also has a built-in method to automatically select the optimal value of β (the soft-thresholding parameter), which is described elsewhere (Russo et al., 2018) and, like the original WGCNA algorithm, it could also be used to disclose correlation networks from proteomics or metabolomics datasets. Pathway enrichment analysis can be run directly from the *webCEMiTool* application, as it interfaces with the Enrichr platform (Kuleshov et al., 2016) which comprises over a hundred gene set libraries, thus facilitating the interpretation and extraction of knowledge from the inferred network.

### Gene Regulatory Networks Permit an Improved Understanding of the Cell’s Transcriptional Circuitry

Gene (transcriptional) regulatory networks, or GRNs, are models that aim at the elucidation of genetic information processing, aiding on the understanding of organism development. A GRN is based on the following elements: TFs, target genes, and their regulatory elements in the upstream region. TFs are identified using computational tools based on sequence homology and through motif conservation across TF families. Each TF can act on the transcription of multiple genes. In the upstream region of each target gene, there exist elements/motifs that are recognized by the TF, and the gene is subsequently transcribed. When located upstream of a gene, these motifs are called *cis*-elements. Identification of *cis*-elements can be performed by biological experiments, such as by chromatin immunoprecipitation (ChIP)-seq methodology (Lee et al., 2006), or computationally by alignment of known motifs or by the identification of novel motifs. The latter are called *de novo* approaches and employ mathematical structures such as hidden Markov models (HMM) (Bailey et al., 2009). Typically, after the identification or discovery of new *cis*-elements, an enrichment analysis is performed using Fisher’s exact test for identification of enriched motifs in the set of upstream regions from target genes.

On the other hand, the prediction of TFs-target genes interactions can be performed using a reverse engineering-based strategy. The top-down approach is particularly suitable in this context and uses information from gene expression datasets to detect expression patterns and then induce a GRN (Hartemink, 2005; Hache et al., 2009). The first models used to infer GRNs were based on the Pearson correlation coefficient but failed to capture non-linear pattern dependencies (as previously addressed). Other approaches were subsequently developed and applied to disclose GRNs in a more robust way, and included regression (Huynh-Thu et al., 2010), mutual information (Margolin et al., 2006a), partial correlations (Wille et al., 2004), and variations of these (Luo et al., 2008; Meyer et al., 2008). Despite each method having its peculiarities, GRNs inferred by diverse techniques usually do not present large differences (de Matos Simoes et al., 2013), and bootstrap analysis could be used to infer more robust GRNs. Another difficulty is the existence of regulation patterns that occur in rare conditions and cannot be easily detected, requiring specific wet-lab experiments for this purpose.

**Figure 4:**
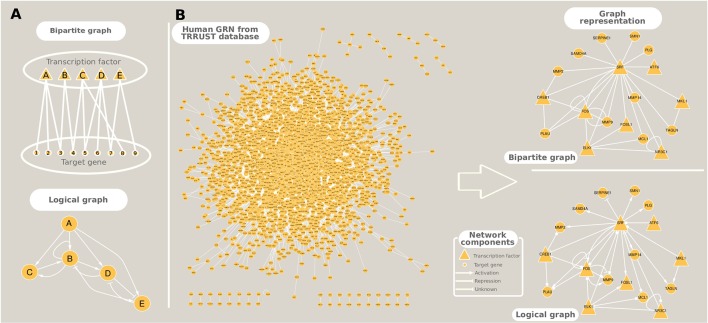
Different ways to represent gene regulatory networks. **(A)** Toy networks exemplifying bipartite and logical (Boolean) graphs. **(B)** A real example of the human gene regulatory network extracted from TRRUST database, and its graphical representation as a bipartite and a logical networks.

The study of gene regulation can take two main paths: i) GRN inference and ii) dynamic modeling, which can be performed either in isolation or in conjunction. We focused on methods that accomplish the first goal, while the latter can be attained using a diverse array of techniques that include Boolean formalism (logical models), Bayesian dynamic networks, and Ordinary Differential Equations (studied elsewhere, e.g., Kaderali and Radde 2008; Naldi et al. 2009; and Chai et al. 2014). The representation of inferred GRNs can be in the form of bipartite graphs which, in contrast to the simple graphs presented in the Introduction and in the construction of co-expression networks, have nodes of two types: TFs or target genes, and edges between them indicate a regulatory interaction (**Table 1**, **Figure 4A**). This type of representation is usually employed to GRNs originated from co-expression relationships because usually no *a priori* information is available about the type of regulation that the TF exerts on the target genes. Logical models, on the other hand, incorporate prior information on gene activation and repression, and the modeling of these relationships permit the capturing of the global dynamic behavior of the regulatory network in a simple fashion. An example of such a network from the human GRN, available in Transcriptional Regulatory Relationships Unraveled by Sentence-based Text mining (TRRUST) database, is shown in **Figure 4B**.

#### User-Friendly Tools for Constructing Gene Regulatory Networks

As seen above, construction of GRNs is based on interaction inference between TFs and target genes, and on the identification of *cis*-elements in the upstream region of target genes. Next, we present user-friendly tools to perform both steps. GRNs inferred based on gene expression patterns are considered of intermediate value because they require improvement and validation with biological experiments. Traditionally, the inference of GRNs has been performed with tools based on command-line or in the R programming language such as ARACNe (Margolin et al., 2006a), but current alternatives include more user-friendly approaches which are listed in **Table 3**. These include an ARACNe implementation in *geWorkbench*, which was listed previously in the correlation network section, and also available are the Cytoscape plugins CyGenexpi (Modrák and Vohradský, 2018), CyNetworkBMA (Fronczuk et al., 2015), GRNCOP2 (Gallo et al., 2011), and iRegulon (Janky et al., 2014) (**Table 3**).

**Table 3:** User-friendly computational tools for inferring gene regulatory networks.

Tool	Description	Platform	Type of data		Reference/URL
			Expression	Promoter	
ARACNe	Creates a network based on Mutual Information	Multi	✓		http://apps.cytoscape.org/apps/aracne; (Floratos et al., 2010)
CyGenexpi	A toolset for identifying regulons and validating gene regulatory networks using time-course expression data	Multi	✓		https://apps.cytoscape.org/apps/cygenexpi; (Modrák and Vohradský, 2018)
CyNetworkBMA	Infers gene regulatory networks from expression measurements using Bayesian Model Averaging	Multi	✓		https://apps.cytoscape.org/apps/cynetworkbma; (Fronczuk et al., 2015)
GRNCOP2	Model-free combinatorial optimization algorithm to infer time-delayed gene regulatory networks from genome-wide time series datasets	Multi	✓		https://apps.cytoscape.org/apps/grncop2; (Gallo et al., 2011)
iRegulon	Allows identification of regulons using motif and track discovery in an existing network	Multi		✓	https://apps.cytoscape.org/apps/iregulon; (Janky et al., 2014)
NetworkAnalyst	Allows establishing TF-target genes and miRNAs-target genes associations.	Webserver	✓		http://www.networkanalyst.ca; (Zhou et al., 2019)
TRRUST	TFs and target genes interactions, and TFs cis-regulatory elements	Webserver	✓	✓	https://www.grnpedia.org/trrust/Network_search_form.php; (Han et al., 2018)
RegNetwork	Genic regulations by TFs and microRNAs	Webserver	✓		http://www.regnetworkweb.org/search.jsp; (Liu et al., 2015)
ORegAnno	Regulatory regions, transcription factor binding sites, etc.	Webserver		✓	http://www.oreganno.org/; (Lesurf et al., 2016)
rSNPBase	Harbors curated information on regulatory SNPs	Webserver		✓	http://rsnp.psych.ac.cn/; (Guo and Wang, 2018)
MEME	Sequence analysis tools for motifs discovery	Webserver		✓	http://meme-suite.org/ (Bailey et al., 2009)

The ARACNe package is based on mutual information index to establish interactions between a pair of genes, such as a TF and a target gene; moreover, this tool employs bootstrapping to generate a consensus and robust network (Margolin et al., 2006b). CyGenexpi is based on an ordinary differential equation model applied on time series data that together with static binding (e.g., ChIP-seq) or information obtained from the literature allows inferring of gene regulatory modules in bacteria (Modrák and Vohradský, 2018). CyNetworkBMA employs a Bayesian model averaging algorithm to infer GRNs with a user-friendly interface and executes network processing on top of R code, which accelerates the inference process by allowing parallel processing (Fronczuk et al., 2015). Additionally, CyNetworkBMA can compute some statistics for the network evaluation, including receiver operating characteristic and precision-recall curves. The package GRNCOP2 has an algorithm based on machine learning with a model-free combinatorial optimization to infer time-delayed GRNs from genome-wide time series datasets (Gallo et al., 2011). The GRNs inference from the iRegulon package is based on analysis of *cis*-regulatory sequences from target genes and performs a genome-wide ranking-and-recovery strategy to detect enriched motifs related to TFs and their optimal sets of direct targets (Janky et al., 2014).

Like other types of biological data, GRNs can be stored on public databases which can be queried by other scientists. In this context, databases that permit storing and downloading of GRNs include TRRUST (Han et al., 2018), RegNetwork (Liu et al., 2015), ORegAnno (Lesurf et al., 2016), and rSNPBase (Guo and Wang, 2018) (**Table 3**). TRRUST database contains information obtained by computational mining and curated TFs-target genes interactions, and about TFs *cis*-regulatory elements in human and mouse. RegNetwork contains information of genic regulations by TFs and microRNAs, also in human and mouse. Similarly, NetworkAnalyst is a webserver that offers an integrated environment to establish TF-target gene and miRNA-target gene interactions (with data sourced from TarBase and miRTarBase). It works by mapping significant genes (such as those found differentially expressed in an RNA-seq experiment) to the corresponding molecular interaction database, and the resulting network can be exported to a Cytoscape-friendly input format. ORegAnno contains information about regulatory regions, TF binding sites, RNA binding sites, regulatory variants, haplotypes, and other regulatory elements for 18 species. Finally, rSNPBase contain information about SNPs on regulatory networks facilitating genetic studies, especially QTL studies.

In the context of *cis*-regulatory elements, this step of GRN inference can be performed either by ChIP-chip experimental approaches or using computational tools from the MEME suite (Bailey et al., 2009), which is a user-friendly web tool (**Table 3**).

### Protein–Protein Interaction Networks Provide an Integrated View of the Proteome’s Organization and Interactions

Proteins are intrinsically involved in every aspect of cellular bioprocesses. Simplistically, they do so by interacting with other proteins and other biocomponents and the resulting interactions may be strong or transient depending on the biological mechanisms at hand. Thus, the analysis of PPIs is a valuable way to study protein complexes, protein function annotation, and states of health and disease (Barabási et al., 2011; Snider et al., 2015).

To begin understanding the emergent characteristics of PPI one has to retrieve interaction data, which can be obtained from high-throughput techniques, interaction databases, or interaction prediction algorithms. The yeast two-hybrid (Y2H) experimental approach verifies the binary interactions between proteins by fusing them to separate *Gal4* TF DNA binding and activating domains (BD and AD, respectively). The principle of the technique relies on the interaction of a protein fused to BD, called *bait*, to the protein fused to AD, called *prey*. If *bait* and *prey* proteins interact, so do BD and AD, restoring the TF activity which is reported in the assay. The Y2H is scalable and can be used to test protein interaction of many proteins in parallel with some automatization (Fields and Song, 1989).

Along with Y2H, the affinity precipitation coupled to mass spectrometry (AP-MS) yields high-throughput interaction data. Affinity purification methods use the specificity of antibody–epitope interaction to co-purify tightly interacting proteins (Bauer and Kuster, 2003). Coupling the purification phase to an identification step using MS provides means to massively generate interaction data. More PPI data can be retrieved from primary databases that store interaction information from experimental data or computational methods for interaction prediction that may involve protein sequence comparison, interologs comparison, protein surface docking, or evolutionary information using co-mutation profiles (Liu et al., 2008; Wiles et al., 2010; Schoenrock et al., 2017).

The nodes in a PPI network are proteins, and an edge is formed between a protein pair when there is evidence of interaction between them (**Table 1**). Interaction evidence may be accompanied by a score or by the qualification of that evidence, which can be set as an edge attribute to weight the support for that interaction. Usually, scores are calculated to assess the confidence in the interaction, i.e., whether the interaction is confirmed by experimental and/or computational methods. The edges in a PPI network are usually undirected, but depending on the specific objective of the reconstruction it could also be set as a directed network (Vinayagam et al., 2011, Vinayagam et al., 2016).

#### User-Friendly Tools for Constructing Protein–Protein Interaction Networks

Many online resources of PPI data are available from different experimental or computational methods and for diverse organisms in varying conditions. The webpage Pathguide^1^ presents a comprehensive list of metabolic pathways and molecular interaction resources available online and indicating if the resources are free to access, whether they follow a systems biology standard for information description and if they are still available. On the PPI section of Pathguide there are 320 listed databases, from which 246 are still online and accessible. On **Table 4** we have listed some general protein–protein database resources. The databases listed are either free or available through academic licensing, with the exception of STRING, which is free to use online, but in order to download the whole database a license must be purchased. The databases are classified as primary, when they gather experimental or literature-based knowledge, or secondary when they gather predicted protein interactions or reflect only a portion of the information available from primary databases (usually performing secondary analyses therein). The DIP database (Xenarios et al., 2000; Salwinski et al., 2004) has experimental interaction information that is curated automatically and manually giving the data high accuracy. STRING, which was briefly presented in the Introduction, is a database that provides experimental and/or predicted protein interaction data for over 5,000 organisms. The IntAct database (Hermjakob et al., 2004; Kerrien et al., 2012) is open-source and maintained by the European Bioinformatics Institute, gathering experimental protein–protein and protein–compound interaction data. With both protein and genetic interaction data from experimental studies, BIOGRID is a freely available primary database (Stark et al., 2006; Chatr-Aryamontri et al., 2017). It is an excellent source of curated experimental data for many model organisms and especially valuable for budding and fission yeasts. The MINT database (Chatr-Aryamontri et al., 2008) provides interaction data derived from the literature and is freely accessible. The I2D database (Brown and Jurisica, 2005, Brown and Jurisica, 2007) is available online and provides data for human PPIs which it imported from primary databases. It can also derive PPI data for other model organisms if they can be mapped to human data. The Center for Cancer Systems Biology (CCSB) provides a primary interaction database named CCSB Interactome Database (http://interactome.dfci.harvard.edu/). The CCSB Interactome Database has experimental binary interaction data for model organisms which can be downloaded and searched freely. APID is a secondary database (Alonso-López et al., 2019) which gathers information from many primary databases, including the Protein Data Bank where protein structures are defined with interacting proteins. As an online web-tool, APID provides the possibility to select interaction properties and interactive mapping of the functional environment of proteins. HuRI, a derivation of the CCSB Interactome Database, is a database with binary PPIs for the human proteome and has three proteome scale protein–protein network reconstructions for the human genome available. Finally, the IID (Kotlyar et al., 2016) database provides tissue-specific interaction data for model organisms and human, harboring both experimental and predicted interactions.

**Table 4:** Online resources for acquiring protein interaction information.

Abbreviation	Name	URL	Availability	Data Source
**DIP**	Database of Interacting Proteins	http://dip.doe-mbi.ucla.edu/dip/Main.cgi	Academic license	Primary
**STRING**	Search Tool for the Retrieval of Interacting Genes/Proteins	http://string-db.org/	License purchase	Secondary
**IntAct**	IntAct Molecular Interaction Database	http://www.ebi.ac.uk/intact	Free	Primary
**BioGRID**	Biological General Repository for Interaction Datasets	http://www.thebiogrid.org/	Free	Primary
**MINT**	Molecular Interaction Database	http://mint.bio.uniroma2.it/	Free	Primary
**I2D**	Interologous Interaction Database	http://ophid.utoronto.ca/	Academic license	Secondary
**CCSB**	Center for Cancer Systems Biology Interactome Database	http://interactome.dfci.harvard.edu/	Free	Primary
**APID**	Agile Protein Interactomes DataServer	http://apid.dep.usal.es/	Free	Secondary
**HuRI**	The Human Reference Protein Interactome Mapping Project	http://interactome.baderlab.org/	Academic license	Primary
**IID**	Integrated Interactions Database	http://iid.ophid.utoronto.ca/iid/Search_By_Proteins/	Academic license	Primary

To analyze interaction data, as for the other two previously discussed network approaches, programmable and graphical user interface options are available. For more advanced users with a programming background, tools such as iGraph and NetworkX allow for automation and processing of large-scale datasets (Csardi and Nepusz, 2006; Hagberg et al., 2013), but user-friendly alternatives also exist, which are compiled in **Table 5**. The first step towards constructing a protein interaction network (PIN) is to get interaction data for proteins of interest. This can be done either by experimentation, as briefly described earlier, and/or by retrieving interaction data from the primary and secondary interaction databases described earlier. Interaction data can be directly downloaded or indirectly retrieved using programs or plugins, as is the case for Cytoscape. On the *Interaction database* category in **Table 5** we list Cytoscape apps that can be used to interrogate and retrieve interaction data from various databases. Bisogenet searches for molecular interaction data from an in-house database, SysBiomics, which integrates data from other interaction databases such as DIP, BIOGRID, BIND, MINT, and IntAct. The searches can be filtered to narrow the interaction space, and protein annotations are retrieved from National Center for Biotechnology Information, Uniprot, KEGG, and GO. The Bisogenet app also includes PIN analysis tools. CyPath2 searches for interaction data from the Pathway Commons integrated BioPAX pathway database. PSICQUIC is a built-in feature of Cytoscape that harbors over 10 million binary interactions from 22 active data providers. The list of active providers of interaction data for PSICQUIC can be seen at the PSICQUIC Registry page^2^. StringApp imports PPI data from STRING with a user provided protein list (or gene, compound, or disease list). Once imported, a matching network of interactions is disclosed, and functional enrichment analysis can be subsequently performed. The previously cited NetworkAnalyst is an online tool for multi-*omics* analysis, also allowing PPI visualization and analysis. It can take a network in standard format, render visualizations and perform network analysis, also receiving a gene list as input to construct an interaction network. Another online option is the Protein Interaction Network Analysis platform (PINA), which generates PINs from a single protein, a list of proteins, a list of protein pairs or two lists of proteins. Networks generated by PINA can be modified with custom data or with different information from other public interaction databases. Lastly, DeDal is a Cytoscape app that embeds data information into the layout of the network, which can facilitate the user in data interpretation (**Table 5**).

**Table 5:** User-friendly computational tools for inferring and analyzing protein interaction networks.

Tool	Description	Category	Reference/URL
Bisogenet	Retrieves interactions associated with input IDs. Sophisticated UI gives links to GO, KEGG, etc.	*Interaction database*	Martin et al., 2010
CyNetSVM	Developed for identification of cancer biomarkers using machine learning approaches.	*PPI-network*	Shi et al., 2017
CyPath2	Pathway Commons (BioPAX L3 database) web service graphical user interface client app.	*Interaction database*	http://apps.cytoscape.org/apps/cypath2
CytoGEDEVO	Pairwise global alignment of PPI or other networks.	*PPI-network*	Malek et al., 2016
CytoMOBAS	Identifies and analyses disease associated and highly connected subnetworks.	*Disease-disease association* *PPI-network*	https://apps.cytoscape.org/apps/cytomobas
DeDal	Applies data dimensionality reduction methods for designing insightful network visualizations.	*PPI-network*	Czerwinska et al., 2015
INTERSPIA	Free online resource for protein interaction comparison between species	Not a Cytoscape app	Kwon et al., 2018
NetworkAnalyst	Free online resource for network construction and analysis	Not a Cytoscape app	Zhou et al., 2019
PathLinker	Reconstructs the interactions in a signaling pathway of interest from the receptors and TFs in a pathway, and can be broadly used to compute and analyze a network of protein interactions.	*PPI-network*	Gil et al., 2017
PEmeasure	Compute links weights and assess the reliability of the links in a network including PPI.	*PPI-network*	Zaki et al., 2013
PEPPER	Find meaningful pathways / complexes connecting a protein set members within a PPI-network using multi-objective optimization.	*Functional module detection*	Winterhalter et al., 2014
PINA	Free online resource capable of PIN construction, filtering, analysis, visualization and management.	Not a Cytoscape app	Wu et al., 2009 Cowley et al., 2012;
PINBPA	Protein-interaction-network-based Pathway Analysis.	*Random walk with restart algorithm*	Wang et al., 2015
PSICQUIC Universal Client	PSICQUIC Web Service Client for importing interactions from public databases.	*Interaction database*	Aranda et al., 2011
stringApp	Import and augment Cytoscape networks from STRING.	*Gene-disease association;* *PPI-network*	Doncheva et al., 2019

PINA, protein interaction network analysis; INTERSPIA, inter-species protein interaction analysis; PINBPA, protein interaction network-based pathway analysis.

For PPI network analysis, besides the previously described online resources, Cytoscape apps can be used. Apps with the *PPI-Network* tag (**Table 5**) can be applied to study the resulting network. CyNetSVM, specifically geared towards identification of cancer biomarkers, takes as input PINs and applies artificial intelligence techniques with gene expression data to aid in the prediction of clinical outcome. CytoGEDEVO is a Cytoscape app that is capable of aligning networks, especially PINs, which can be used to study the evolution and conservation of proteins interactions. A different approach on comparison of PPIs is used by the online application INTERSPIA, which is freely available. INSTERSPIA can identify interacting proteins in a user-specified list and disclose similar interaction patterns across multiple species. PE-measure, another Cytoscape app, can be used to confirm protein interactions in a network based on its structure, also helping users to identify spurious interactions. Further analysis in PPI networks can be achieved using other tools in Cytoscape. PEPPER, for instance, identifies protein complexes or pathways that are highly condensed using a gene set list as input, helping to integrate information such as protein connections with proteins on the gene set list that are involved in a particular phenotype change, e.g., disease, by finding functional modules. PINBPA is another app that aids in module discovery and is especially suited to integrate GWAS data into protein–protein networks, which can help identify enriched sub-networks and prioritize relevant genes. In the following section we return to the identification of modules in networks in general using algorithms that rely only on the network topology. Finally, PathLinker, a Cytoscape app, can infer signaling networks from PPI networks by computing short paths in a PIN between receptor proteins, as source nodes, and target proteins, as TFs.

### A Primer on Network Analysis and Visualization

Once a network of interest is attained, downstream analyses are warranted to extract relevant information and gain knowledge from the reconstruction. These analyses can be broadly divided into *knowledge extraction* and *visualization* steps. There are many methods to evaluate a network and leverage knowledge to help guide interpretation, and this usually begins by exploring local and global interactions within the network. Metrics such as modularity, degree distribution, and other centrality measures are commonly applied to assist in the identification of important or influential nodes in a network (Freeman, 1978; Jeong et al., 2001; Barabási, 2016) (see **Box 1**). Cytoscape has the built-in plugin *NetworkAnalyzer* (Assenov et al., 2008) that computes many centrality metrics, and these can be extended by the Centiscape plugin, which implements ten centrality indexes (Scardoni et al., 2009). Gephi also provides built-in methods to calculate betweenness, eigenvector, and closeness centrality measures, while bridging centrality can be calculated via a third-party plug-in (Bastian et al., 2009). Different centrality methods will usually arrive at distinct rankings of important nodes, which is not unexpected since in order to establish importance each method takes into account different aspects of the data. Betweenness centrality, for instance, emphasizes the importance of a node by considering its contribution in allowing information to pass from one part of the network to the other (thus, a global measure of centrality), while degree centrality simply counts the number of connections between a node and its direct neighbors (thus, a local measure of centrality). For some applications, a combination of centrality metrics may be more appropriate, as has been suggested for metabolic network analysis (Rio et al., 2009). In **Box 1** we present a comparison between selected centrality measures using a toy network, but an exhaustive evaluation is out of the scope of the current work, and efforts have been made to categorize and describe the various centrality indexes, such as the CentiServer online resource (http://www.centiserver.org) (Jalili et al., 2015), which harbors 232 measures of centrality in its last 2017 update, allowing users to input a network and calculate 55 centralities indexes in an interactive web-based application. The use of centrality measures in biological networks dates back to 2001, when Jeong et al. (2001) postulated the ‘centrality-lethality rule’ using a yeast PIN, and found that the most highly connected proteins in the fungi’s cellular network were those more important for its survival, establishing a connection between centrality (a graph-theoretical concept) and essentiality (a biological concept).

**Figure 5:**
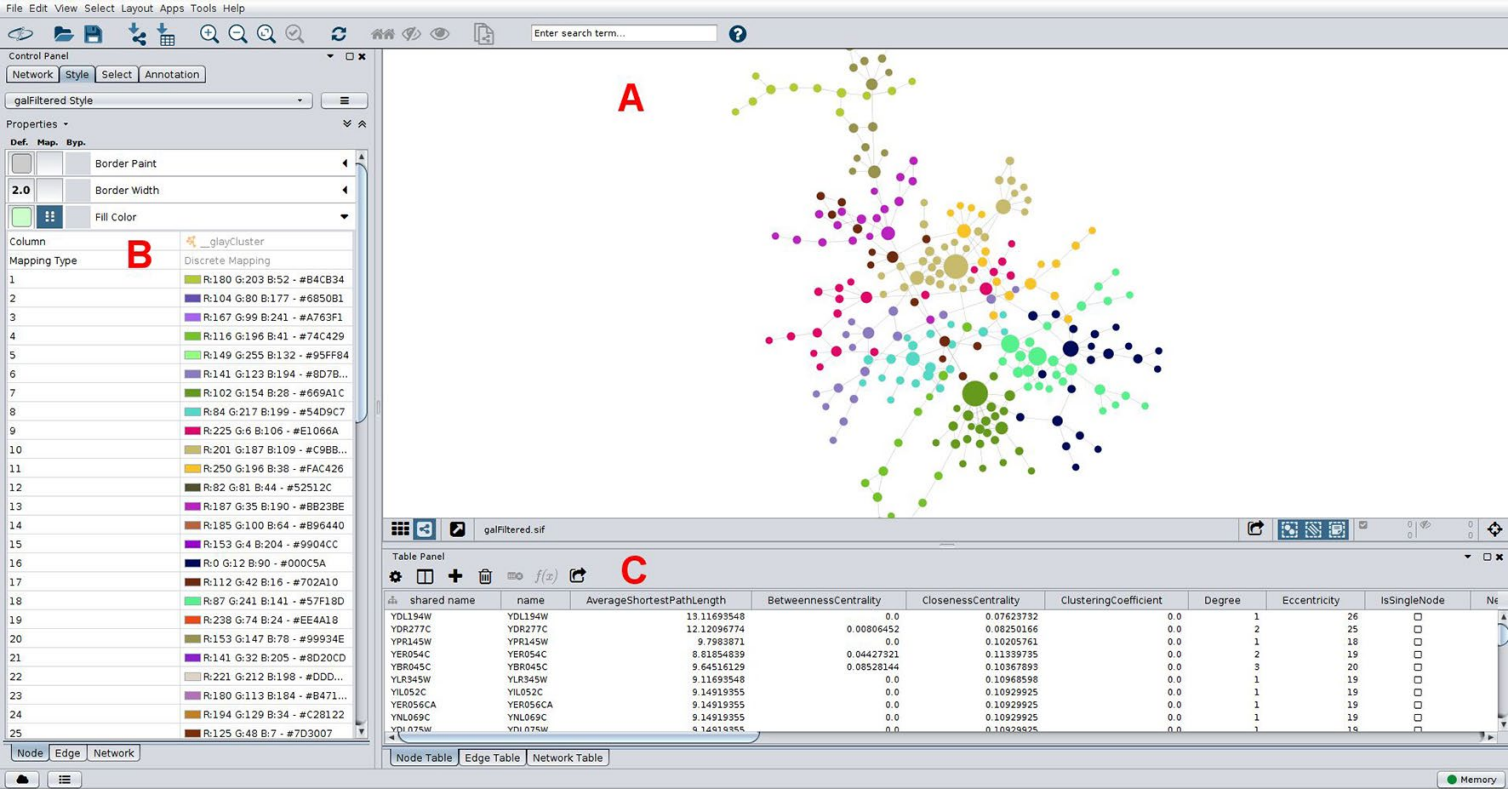
Typical network analyses performed using Cytoscape. A network of yeast protein interaction data is presented **(A)**, with node size scaled with betweenness centrality, which help in straightforward identification of important nodes in this network. Nodes are colored according to its membership to a community as determined using the Girvan-Newman fast greedy algorithm implementation in the *clusterMaker* plugin (Morris et al., 2011). Colors for each community were chosen automatically using a color-generating function and a discrete mapping, with modules numbered sequentially in the left column shown in **(B)**, and colors (in RGB and hex formats) on the right. Properties of nodes are shown below in **(C)**, including some centrality measures. These can be downloaded in-whole as a table for downstream analyses. The network is arranged according to a force-directed layout algorithm.

Biological networks usually display internal structures that can be identified as subnetworks in modularity analysis (Blondel et al., 2008), which present as densely connected regions, and the disclosed modules can be visually inspected by applying, for instance, the *qgraph* approach (Epskamp et al., 2012) (**Figure 2D**). Modularity (or *Q*) is used as a metric for defining the partitioning of a network and increases its value with increasing network community structure (Newman, 2006). The maximum modularity for a network is *Q* = 1, but in practice values for networks with strong community structure are typically in the range of 0.3-0.7 (Newman and Girvan, 2004). Many module detection techniques have been developed in the recent years and broadly divide into clustering, decomposition, and biclustering methods, which have been subject of recent reviews (Saelens et al., 2018 Rahiminejad et al., 2019). Another use of this approach is to infer biological functions using the guilty-by-association principle, where the role of an uncharacterized gene (or protein) can be predicted by considering the broad functions of the genes with which it clusters in a modularity analysis. As an example, groups of co-expressed genes have a greater chance of being functionally coupled, either by participating in a common biological pathway or by a shared regulatory mechanism, such as an upstream regulator. In this way, novel hypotheses about gene function are generated which can be subsequently explored using as basis a co-expression network. This strategy has successfully led to the identification of novel schizophrenia risk genes, where a co-expression gene set enriched for protein-coding genes associated with the disease was disclosed (Pergola et al., 2017). As was the case for centrality metrics, both Gephi and Cytoscape offer modules to perform clustering analysis, and a Cytoscape example is shown in **Figure 5**. Gephi implements natively the Louvain algorithm, that finds modules by exploring the idea of increasing the network modularity in two phases: first, local modularity gains when neighboring nodes are included in the same cluster in an iterative fashion, which leads to local modularity maxima; second, by considering the disclosed modules from the first phase as communities and aggregating these communities iteratively (forming meta-communities) until attaining a new modularity maximum which cannot be increased further (Blondel et al., 2008). The efficiency of this algorithm allows its application to very large networks on the order of millions of nodes, one of the reasons why it has gained widespread adoption, with almost 9,000 citations (Blondel et al., 2008), including its application to disclose modules related to hepatic dysfunction (Soltis et al., 2017) and cancer (Ajorloo et al., 2017). Other clustering methods available in Gephi through third-party plugins are the Leiden (Traag et al., 2019) and the Girvan-Newman algorithms (Girvan and Newman, 2002). Girvan-Newman works by sequentially removing edges from the network until reaching a maximum modularity, and the nodes that remain connected in the resulting network represent the communities. It has been applied to a wealth of problems (accumulating over 11,000 citations), including to the successful recovery of communities of taxonomically-related organisms using protein sequence data as input (Andrade et al., 2011), but has the drawback of scaling cubically with the number of nodes in its worst case scenario, which limits its use to networks having not more than a few thousand nodes (Girvan and Newman, 2002; Rahiminejad et al., 2019). The Leiden method appeared more recently and claims to improve the quality of the disclosed modules compared to Louvain’s method, as well as address some of its shortcomings (Traag et al., 2019). Other clustering methods are available through Cytoscape packages such as *clusterMaker* (Morris et al., 2011) and *CytoCluster* (Li et al., 2017b), with the latter implementing six clustering methods including OH-PIN. In contrast to the previous algorithms that only detect modules containing non-overlapping elements, OH-PIN discloses overlapping clusters typical of many biological networks, such as enzymes that catalyze reactions across multiple pathways.

Once a network is constructed and analyzed from a topological standpoint using the previous approaches, several layout algorithms can be employed to generate visualizations of the network. While different visualization strategies do not alter the connectivity patterns between nodes, they aid during the identification of influential nodes and communities, while also allowing the organization of the network according to specific properties it may present, such as an underlying node hierarchy. Many layout algorithms are constrained by network size and can perform poorly (consuming extensive memory and CPU) when applied to the ordering of very large networks. Both Gephi (Bastian et al., 2009) and Cytoscape (Shannon et al., 2003) have a plethora of built-in visualization algorithms. In order to arrive at a suitable and pleasant network visualization a number of trial-and-error is involved, not only by qualitatively selecting layout algorithms (which can be coupled in sequence), but also by experimenting with different parameterizations schemes. Force-based algorithms are widely used to arrange networks and follow the general rule that linked nodes attract each other and non-linked nodes are mutually repelled, with inspiration from mechanical forces such as tension and compression acting through a spring, temperature gradients, or even electromagnetic forces. These methods rely only on the topology of the graph in order to arrange the nodes. Consequently, networks laid out according to force-directed strategies usually present similar edge lengths which have a low number of crossings, resulting in an aesthetically pleasing visualization. In Cytoscape, force-directed-based algorithms include the compound spring embedder and prefuse force-directed spring layout, while Gephi implements ForceAtlas2, Fruchterman-Reingold, Yifan-Hu, and OpenOrd. OpenOrd is particularly suitable for large graphs, scaling well for networks over 1 million nodes, and can be followed by the Yifan-Hu layout in order to produce appealing visualizations in such large networks (Pavlopoulos et al., 2017). Both Gephi and Cytoscape can expand their repertoire of layout methods using third-party plugins, such as the proprietary yFiles plugin for Cytoscape which offers nine options for network layout, many of which are multi-purpose such as the force-directed organic (which works well for large graphs) and orthogonal layouts (best applicable to medium-sized networks, routing edges orthogonally), as well as the hierarchic (useful for portraying precedence relationships) and circular layouts (producing star and ring topologies that are useful for visualization of regulatory relationships).

## Networks, Networks Everywhere: Health and Disease From a Global Standpoint

**Figure 6:**
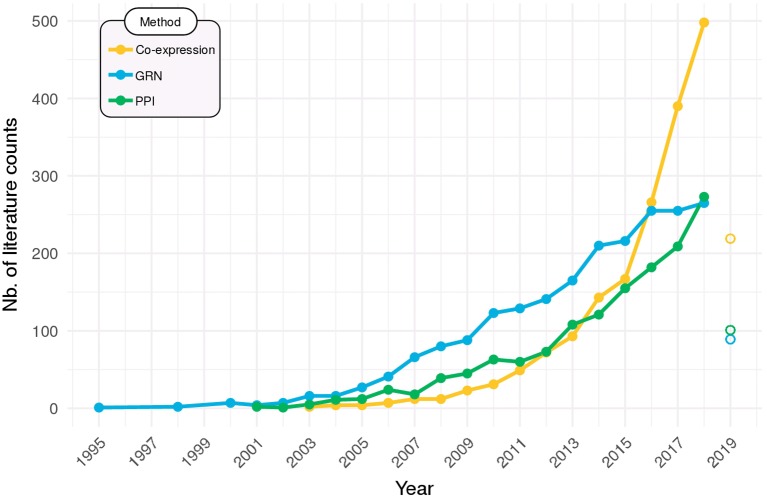
Network methods on the rise. Searches in PubMed (http://ncbi.nlm.nih.gov/pubmed) were performed to identify the all-time use of co-expression networks (query: “co-expression network” OR “coexpression network”), gene regulatory networks (GRN; query: “gene regulatory network”), and protein–protein interaction networks (PPI; query: “protein–protein interaction network”). Data for 2019 is partial (up to March) and are displayed as open points.

Networks are now widely employed to help make sense of high-throughput *omics* data. **Figure 6** shows that usage of the networks methods that were covered in this Review is on the rise in the scientific literature. Particularly in the last 5 years, there has been a steep increase in their adoption, especially for co-expression networks, which can be partly due to the falling of sequencing costs, but also to the recent availability of some of the more user-friendly tools that were put available and reviewed herein.

Integrative approaches are particularly suitable for the study of diseases, as they are hardly the effect of single perturbations. These networks allow the identification of associations between the measured components as well as identifying communities (or modules) that could mediate a link between normal and diseased states, including regulatory interactions. Applications of correlation networks include hub genes identification in several diseases such as cancer (Oh et al., 2015), chronic fatigue syndrome (Presson et al., 2008), diabetes (Keller et al., 2008), and in the multivariate disease autism (Voineagu et al., 2011). The use of networks in the context of the neglected tropical disease leishmaniasis was also recently reviewed (Veras et al., 2018). Also performed were the stratification of breast cancer subtypes using human plasma metabolomics (Fan et al., 2016), the study of extracellular proteins in serum to disclose information on human disease states (Emilsson et al., 2018), and the evaluation of coordinated expression patterns in different brain regions in Alzheimer’s disease (Wang et al., 2016a). These many studies revealed important pathways and networks of interconnected bioelements that associate with health and disease phenotypes. Co-expression and correlation networks were also used to understanding the immune response of humans to vaccination, disclosing vaccine-induced transcriptional signatures that correlated to protection (Nakaya et al., 2015; Li et al., 2017c), and have also been derived from multi-*omics* data to the understanding and tackling of disease complications from diabetes-tuberculosis comorbidity, where a correlation network constructed from whole-blood gene expression and plasma cytokine measurements was obtained (Prada-Medina et al., 2017).

Finally, disease-disease association uses the information of disease-modules in order to identify common nodes (proteins, genes, metabolites) between diseases which can help pinpoint disease comorbidity or predisposition between conditions. This approach can potentially accelerate drug design since drugs that target interactions that are common between conditions could have a better treatment impact (Barabási et al., 2011). These methods were widely employed to construct disease-disease and gene-disease networks (Serão et al., 2011; Li et al., 2017a; Wiredja et al., 2017; Dong et al., 2018; Liu et al., 2019; Zhang et al., 2018).

While co-expression and PPI networks are tightly related, they are both under the control of regulatory elements, thus the importance of GRNs. Environmental stimuli, pathogen exposure and other disease statuses can trigger a myriad of responses in a cell, including the cascade signals that are recognized by TFs, which in response modulate gene expression. Due to the specificity of GRNs for the conditions of interest, there are multiple GRNs that were generated from specific conditions, such as tissues, environments, pathologies, and the combination of these factors (Guan et al., 2012; Emmert-Streib et al., 2014). This availability of networks from specific conditions can be used to support other studies with similar conditions or used to improve GRNs for other species. In this context, GRNs can be used in health as maps and biomarkers to characterize genetic perturbations associated to rare hereditary variants such as SNPs in the regulatory region of a disease-related gene of interest (Guo and Wang, 2018).

## Conclusions

A variety of tools are available to support the construction of biological networks from *omics* data. Although user-friendliness is usually not a top priority for developers, it can be readily attained with the help of excellent frameworks such as Cytoscape, for which a multitude of plugins are available that permits greatly expanding the capacities of the software beyond its original scope. Also, webserver versions of hitherto command-line only software are increasingly being published. We expect that user empowerment through the breaking of barriers imposed by programming language requirements will allow further adoption of network strategies and accelerate the extraction of knowledge and insights from biological data.

## Author Contributions

PR conceived the review scope and outline. AQ, KF, LA, NL, and PR wrote the review. PR edited the final version with support from the other authors. All authors read and approved the final version.

## Funding

NL received financial support from the Conselho Nacional de Desenvolvimento Científico e Tecnológico (CNPq), Brazil [Universal 28/2018; grant protocol 427183/2018-9]. LA received a postdoctoral fellowship from the Coordenação de Aperfeiçoamento de Pessoal de Nível Superior (CAPES). AQ acknowledges funding from Fundação Oswaldo Cruz (INOVA - Process VPPIS-001-FIO-18-45). Publication fees were defrayed by Fundação Oswaldo Cruz. The funders had no role in study design, analysis, decision to publish, or preparation of the manuscript.

## Conflict of Interest

The authors declare that the research was conducted in the absence of any commercial or financial relationships that could be construed as a potential conflict of interest.

## Footnotes

1 http://www.pathguide.org; the webpage is maintained by Dr. Gary Bader at the University of Toronto.

2 Available at http://www.ebi.ac.uk/Tools/webservices/psicquic/registry/registry?action=STATUS .
